# Asymmetric Dimethylarginine Is a Well Established Mediating Risk Factor for Cardiovascular Morbidity and Mortality—Should Patients with Elevated Levels Be Supplemented with Citrulline?

**DOI:** 10.3390/healthcare4030040

**Published:** 2016-07-08

**Authors:** Mark F. McCarty

**Affiliations:** Catalytic Longevity, 7831 Rush Rose Dr., Apt. 316, Carlsbad, CA 92009, USA; markfmccarty@gmail.com; Tel.: +1-760-216-7272; Fax: +1-760-704-6379

**Keywords:** cardiovascular, asymmetric dimethylarginine, nitric oxide synthase, citrulline, tetrahydrobiopterin, folic acid, statins, spirulina, NADPH oxidase, Alzheimer’s disease

## Abstract

The arginine metabolite asymmetric dimethylarginine (ADMA) is a competitive inhibitor and uncoupler of endothelial nitric oxide synthase (eNOS), an enzyme that acts in multifarious ways to promote cardiovascular health. This phenomenon likely explains, at least in part, why elevated ADMA has been established as an independent risk factor for cardiovascular events, ventricular hypertrophy, and cardiovascular mortality. Fortunately, the suppressive impact of ADMA on eNOS activity can be offset by increasing intracellular arginine levels with supplemental citrulline. Although the long-term impact of supplemental citrulline on cardiovascular health in patients with elevated ADMA has not yet been studied, shorter-term clinical studies of citrulline administration demonstrate effects suggestive of increased NO synthesis, such as reductions in blood pressure and arterial stiffness, improved endothelium-dependent vasodilation, increased erection hardness, and increased ejection fractions in patients with heart failure. Supplemental citrulline could be a practical option for primary or secondary prevention of cardiovascular events and mortality, as it is inexpensive, has a mild flavor, and is well tolerated in doses (3–6 g daily) that can influence eNOS activity. Large and long-term clinical trials, targeting patients at high risk for cardiovascular events in whom ADMA is elevated, are needed to evaluate citrulline’s potential for aiding cardiovascular health.

## 1. Asymmetric Dimethylarginine—A Competitive Inhibitor of Endothelial Nitric Oxide Synthase

Asymmetric dimethylarginine (ADMA) is produced within cells by dimethylation of arginine within intact proteins by a class of enzymes known as protein arginine *N*-methyltransferases (PRMT); subsequent proteolysis releases free ADMA [1]. “Asymmetric” refers to the fact that one of the nitrogens in the guanidino head group of arginine is dimethylated, whereas the other remains unmethylated. Its chemical relatives symmetric dimethylarginine (SDMA—in which each of the guanidino nitrogens is monomethylated) and *N*-monomethylargine (NMMA) are produced in a comparable fashion. There are six known isoforms of PRMT; type I enzymes, of which PRMT-1 has the broadest activity, are responsible for asymmetric dimethylation (and hence promote ADMA synthesis), whereas type II enzymes, most notably PRMT-5, catalyze symmetric dimethylations [2].

ADMA and NMMA can be degraded by the widely expressed enzyme dimethylarginine dimethylaminohydrolase (DDAH), of which there are two isoforms; DDAH-1 is the predominant form in the liver and in kidney tubules, whereas DDAH-2 contributes importantly to the DDAH activity of vascular endothelium and smooth muscle, reflecting the ability of nitic oxide/cGMP to increase its expression [3,4,5,6,7,8]. DDAH appears to be responsible for about 80% of ADMA clearance, and hence its activity is a crucial determinant of ADMA levels [9]. A mitochondrial enzyme expressed primarily in the kidneys, alanine-glyoxylate aminotransferase 2, also can catabolize ADMA [10]. SDMA is removed by renal clearance, and a minor portion of ADMA is cleared in this way. Within endothelial cells, oxidative stress tends to boost ADMA levels both by increasing expression of PRMT-1, and by inhibiting the activity of DDAH [11,12,13,14,15]. As is well-known, oxidative stress, much of it stemming from NADPH oxidase complexes, is a key feature of the endothelial dysfunction typically seen in diverse cardiovascular disorders [16].

ADMA has a high affinity for the active site of endothelial nitric oxide synthase (eNOS), competing with arginine in that regard. Although intracellular arginine levels typically are considerably higher than eNOS’s K_m_ for arginine, high-normal levels of ADMA are sufficiently high to diminish eNOS activity via competition with arginine [17]. This reflects the fact that ADMA is concentrated within endothelial cells by active transport, such that its intracellular level is nearly an order of magnitude higher than its plasma level [18]. Moreover, ADMA-bound eNOS is uncoupled, such that it generates superoxide rather than nitric oxide [19,20,21]. A vicious cycle mechanism can be envisioned, whereby ADMA promotes superoxide production by eNOS, and the resulting oxidative stress up-regulates ADMA levels. NMMA can also competitively inhibit eNOS, but this is likely to be of less physiological significance, since plasma concentrations of NMMA are only about one-fifth as high as those of ADMA, and its affinity for eNOS is slightly lower [18,22]. While SDMA does not influence eNOS activity directly, it does compete with arginine for transport into cells, as ADMA does [23]. Hence, all three of these agents have the potential to suppress endothelial production of nitric oxide (NO).

Given the central role of NO in maintenance of cardiovascular health [24,25], it is logical to suspect that relatively elevated levels of ADMA and SDMA could increase cardiovascular risk. ADMA levels tend to be elevated when renal function is compromised, or when DDAH activity is suboptimal. Notably, DDAH is susceptible to inhibition by oxidative stress, an effect mediated, at least in part, by 4-hydroxynonenal, a degradation product of peroxidized fatty acids [13,14].

## 2. ADMA Is an Established Independent Risk Factor for Cardiovascular Morbidity and Mortality

A number of prospective epidemiological studies have concluded that moderately elevated ADMA levels are associated with notably increased risk for coronary events and cardiovascular mortality in the general population [26,27,28,29,30,31,32,33,34,35]. These associations persist in multiple regression analyses accounting for covariant risk factors such as blood pressure (these associations are less clear in current smokers, likely because smoking tends to lower ADMA levels by inducing endothelial DDAH activity [30]). In a recent meta-analysis of pertinent studies, risk ratios for the top third versus the bottom third of serum ADMA were found to be 1.42 (95% CI 1.29–1.56) for cardiovascular disease, 1.39 (1.19–1.62) for coronary heart disease, and 1.60 (1.33–1.91) for stroke [35]. These risk ratios were at least as high in studies focusing on patients with pre-existing cardiovascular or renal disease. Additionally, in patients with normal or compromised renal function, ADMA levels correlate with left ventricular mass independent of blood pressure—an observation that may reflect the role of myocardial eNOS activity in countering ventricular hypertrophy and promoting efficient diastolic relaxation [36,37,38,39,40,41]. Some, though not all studies [42], find that elevated ADMA also predicts poorer outcomes in patients with existing heart failure [43,44,45]. ADMA has also emerged recently as a likely mediator of the increased cardiovascular risk associated with systemic autoimmune syndromes, such as rheumatoid arthritis or Sjogren’s syndrome, in which ADMA correlates inversely with coronary flow reserve [46,47,48].

Hence, ADMA has emerged as an independent risk factor for cardiovascular morbidity and mortality. Although ADMA may function as a marker for renal failure and perhaps endothelial oxidative stress—both of which can play a mediating role in cardiovascular disease [49]—there is good reason to suspect that elevated ADMA also plays a mediating role in this regard, in light of its impact on eNOS activity.

## 3. Supplemental Citrulline—A Practical Antidote to ADMA

Fortunately, there may be a simple antidote to the adverse physiological effects of ADMA—the amino acid citrulline [50,51,52]. Citrulline is produced from arginine by NOS activity, and it is rapidly reconverted to arginine within cells. Since citrulline is readily transported into cells, it can be employed to boost intracellular arginine levels; as it employs a different transporter than the cation arginine, ADMA and SDMA cannot suppress its intracellular uptake. Paradoxically, citrulline supplementation does a more effective job of increasing systemic arginine levels than does arginine supplementation, as orally administered arginine is susceptible to rapid degradation (to ornithine and urea) by intestinal and hepatic arginase activity [50,51,52]. Furthermore, the physiological impact of arginine supplementation tends to fade over time, as such supplementation tends to induce increased arginase activity. In contrast, arginase does not degrade citrulline, and indeed citrulline functions as a competitive inhibitor of this enzyme [51,52].

If, as seems likely, moderately elevated ADMA plays a mediating role in cardiovascular disorders, it would be logical to propose that ADMA levels should be screened in the general population, and citrulline supplementation recommended for those with higher levels. This may be practical, as citrulline is not very expensive in multi-gram daily doses, is well tolerated, and has a very mild flavor, making it feasible to incorporate citrulline into functional foods and beverages. Indeed, the richest natural source of citrulline is watermelon juice [53,54] (in contrast, arginine has an acrid flavor).

However, relatively few clinical studies have evaluated the effects of supplemental citrullline, and none have been sufficiently large and long to determine the impact of such supplementation on long-term endpoints such as cardiovascular events or mortality. Moreover, no studies have studied citrulline in people pre-determined to have high ADMA levels. Nonetheless, when administered in oral doses of 3–6 grams daily, supplemental citrulline has been shown to exert various effects suggestive of NO-mediated cardiovascular protection: Reductions in systolic and diastolic blood pressure and in arterial stiffness, improvements in endothelium-dependent vasodilation, improvements in erection hardness in patients with erectile dysfunction, increased left and right ejection fractions in patients with heart failure, and an improved clinical course in sickle cell patients [50,55,56,57,58,59,60,61,62,63,64,65].

## 4. Supplemental Citrulline Should Be Studied in High-Risk Patients with Elevated ADMA

What is now needed is a large and lengthy trial of citrulline supplementation enrolling patients judged at high cardiovascular risk (post-MI, for example) who have been pre-determined to have relatively high serum levels of ADMA—in the third or fourth quintile, perhaps. A successful outcome in such a study, and subsequent comparable studies, would suggest the desirability of measuring ADMA in the general patient population—much like other established risk factors such as LDL cholesterol and blood pressure are measured—and of recommending supplemental citrulline for those patients found to have elevated ADMA levels. The fact that citrulline is an unpatentable nutraceutical, as opposed to a patented drug, means that it will be more difficult to organize such studies, and may explain why no such studies have already been conducted, despite that fact that ADMA is now quite well documented as a robust and independent cardiovascular risk factor.

A limited amount of evidence suggests that elevated SDMA may also be a cardiovascular risk factor [66,67,68]. If so, it is notable that citrulline can bypass SMDA’s impact on arginine uptake, and hence may counteract the adverse physiological effects of SDMA.

## 5. Recoupling of eNOS also Requires Tetrahydrobiopterin

Unfortunately, provision of optimal intracellular levels of arginine does not guarantee that eNOS will regain its coupled activity, as this enzyme can also become uncoupled owing to suboptimal levels of its essential cofactor tetrahydrobiopterin (BH4) [69,70,71]. Peroxynitrite, which arises in oxidatively-stressed endothelial cells from the spontaneous interaction of nitric oxide and superoxide, can readily oxidize BH4 to dihydrobiopterin (BH2), which competes with BH4 for binding to eNOS; in its BH2-bound form, or in the absence of BH4, eNOS is uncoupled [70,71]. Remarkably, either arginine or ADMA increase eNOS superoxide generation when BH4 is absent [72].

Although it would be theoretically possible to promote eNOS recoupling with supplemental BH4, the instability and high cost of this compound render such an approach problematic. Supplementing with high-dose folic acid represents a more practical strategy. In high doses—an order of magnitude or more higher than nutritionally essential intakes—folic acid tends to boost endothelial levels of BH4 for two reasons. First, the reduced metabolites of folic acid formed within cells are potent scavengers for peroxynitrite-derived radicals, and hence tend to protect BH4 from oxidation [73,74,75]. Secondly, folate dose-dependently boosts endothelial expression of dihydrofolate reductase, which not only functions in folate metabolism, but also efficiently re-reduces BH2 to BH4, boosting the ratio of BH4 to BH2 and hence promoting recoupling of eNOS [76,77,78,79]. Recoupling of eNOS with high-dose folate has been demonstrated in both clinical and rodent studies [74,80,81,82,83]. Intriguingly, several decades ago, cardiologist Kurt Oster reported that folate supplementation at 40–80 mg daily provided marked benefit in angina and intermittent claudication, without side effects; unfortunately, he did not attempt controlled trials, and his claims were largely ignored [84,85,86]. However, his claims appear consistent with the results of a controlled clinical study, in which an acute dose of 30 mg folic acid was found to enhance the coronary flow response to adenosine in patients with ischemic heart disease [81]. Concurrent oral supplementation with high-dose vitamin B12—e.g., 1 mg daily—could correct any pre-existing B12 deficiency, and hence alleviate concerns that high-dose folate administration could mask the early signs of pernicious anemia [87,88].

Hence, concurrent supplementation with supraphysiological doses of folate (and vitamin B12) could be expected to insure that citrulline administration would promote optimal coupled activity of eNOS in patients with elevated ADMA.

## 6. Inhibition of NADPH Oxidase May Decrease ADMA Levels

Strategies for lowering elevated ADMA levels might also be useful. A recent meta-analysis concluded that statins are modestly useful in this regard [89]. Since these drugs have the potential to down-regulate endothelial NADPH oxidase activity by suppressing isoprenylation of Rac1 [90,91], it is reasonable to suspect that protection of DDAH activity from oxidative stress contributes to this effect.

Importantly, the superoxide generated by NADPH oxidase can quench NO, resulting in the production of highly reactive peroxynitrite. As noted, this latter compound, via oxidation of the cofactor tetrahydrobiopterin, can “uncouple” eNOS. Hence, suppression of NADPH oxidase activity could be expected to decrease ADMA levels, prevent the quenching of NO, and oppose uncoupling of eNOS—all of which would promote effective NO bioactivity. Moreover, NADPH oxidase activity has pro-inflammatory effects on endothelial function, mediated via NF-kappaB, that are independent of its impact on NO bioactivity [92,93,94].

It would be of interest to determine whether serum bilirubin levels correlate inversely with ADMA, as intracellular free bilirubin functions physiologically as an inhibitor of NADPH oxidase complexes in low nanomolar concentrations [95,96,97,98]. Numerous prospective studies have associated increased serum bilirubin with lower cardiovascular risk [99,100]. Phycocyanobilin (PhyCB), a chromophore of edible cyanobacteria, such as spirulina, which is similar in structure to bilirubin, may have the potential to function clinically as a mimetic of bilirubin’s antioxidant activity, and hence conceivably could modulate ADMA levels [101,102]. It should be technically feasible to combine citrulline, spirulina, and folate in functional foods.

## 7. Additional Potential Benefits of Citrulline—Preventing Alzheimer’s

While citrulline-mediated support of effective eNOS activity in the brain microvasculature would seem likely to reduce risk for both stroke and vascular dementia, there is also reason to suspect that it might counteract Alzheimer’s pathology—in part by suppressing production of amyloid beta. Case-control studies have reported increased plasma levels of ADMA, and reduced levels of NO metabolites, in patients with Alzheimer’s disease (AD) [103,104] (curiously, levels of ADMA in cerebrospinal fluid tend to be decreased). While it is conceivable that ADMA here is serving only as a marker for vascular pathology that might work in other ways to promote development of AD, rodent and cell culture studies suggest that microvascular eNOS activity may function to restrain the expression of both amyloid precursor protein and beta-secretase in brain parenchyma and vasculature—thereby suppressing production of amyloid beta—and also to down-regulate microglial activation. Genetically-altered mice heterozygous for eNOS deficiency develop regions of brain hypoperfusion, brain microinfarcts, cerebral amyloid angiopathy associated with vascular amyloid beta deposition, and impaired cognitive function—defects not seen in mice with full eNOS activity [105]. Studies with mice homozygous for eNOS loss also show increased brain expression of APP and beta-secretase, increased amyloid beta deposition in the brain and cerebral vasculature, and increased microglial activation—all of which could be mitigated when the mice were treated with nitroglycerin [106,107,108,109]. Treatment of a human neuron-derived cell line with ADMA boosted its secretion of amyloid beta [110]. In autopsy studies evaluating AD patients, the number of eNOS-positive brain capillaries correlated inversely with the presence of neurofibrillary tangles and senile plaques [111,112]. In normal rats, brain neuronal levels of caveolin-rich lipid rafts high in APP and the C99 membrane fragment of APP stemming from beta-secretase activity tend to increase markedly during aging; three months of citrulline supplementation in aging rats tends to reverse this change [113]. Asif and colleagues, reviewing the pertinent literature, conclude that “ADMA might represent a pathophysiological pathway linking the presence of vascular risk factors with the onset and progression of cognitive decline and dementia” [114].

In light of these findings, it is reasonable to suspect that, in individuals with elevated plasma ADMA levels, citrulline supplementation might slow the onset or progression of AD. Studies testing the impact of supplemental citrulline in AD model mice have been recommended [113]. However, eNOS activity in AD cerebral vasculature can be compromised not only by ADMA, but also by oxidative stress stemming from activated NADPH oxidase, leading to eNOS uncoupling [115]. Amyloid beta activates NADPH oxidase in the cerebral microvaculature via the C36 receptor [115]. Hence, antioxidant measures such as statins or PhyCB might complement citrulline in promoting effective cerebrovascular eNOS activity. However, whether high-dose folate could promote eNOS recoupling in the cerebral microvasculature is questionable, as the folate uptake of these cells, constituting a portion of the blood-brain barrier, appears to be maximized at physiological blood levels of folate [116].

## 8. Conclusions

ADMA has emerged as an independent risk factor for cardiovascular events and mortality; it also has been linked to increased risk for ventricular hypertrophy and diastolic dysfunction. While increased ADMA may serve as a marker for oxidant stress and reduced glomerular filtration, there is also good reason to suspect that its inhibitory and uncoupling impact on protective eNOS activity is at least partially responsible for its association with adverse cardiovascular outcomes. Inasmuch as supplemental citrulline can raise tissue arginine levels efficiently and thereby offset ADMA-mediated inhibition of eNOS, it has the potential to support cardiovascular health in patients with elevated ADMA levels. Evidently, large, long-term clinical trials, targeting patients judged at high risk for cardiovascular events whose ADMA levels are elevated, are needed to assess the protective potential of supplemental citrulline as an “antidote” to ADMA. Citrulline powder is inexpensive and mild flavored, so its use in secondary or primary prevention of cardiovascular disorders should be reasonably practical; 3–6 g daily may be needed for optimal efficacy in this regard. Concurrent supplementation with supraphysiological doses of folic acid, by helping to insure optimal endothelial levels of BH4, could help to insure that citrulline administration restores coupled eNOS activity. Measures which suppress endothelial NADPH oxidase activity—statins and possibly PhyCB—could be expected to decrease ADMA levels while acting in other ways to support efficient NO bioactivity. The possibility that elevated ADMA contributes to the progression of Alzheimer’s disease by suppressing brain-protective effects of eNOS in the cerebral microcirculation merits further study, and might point toward a role for supplemental citrulline in minimizing dementia risk.
